# Vancomycin Sensitization in is Contingent on Limited Metabolic Flux

**DOI:** 10.1021/acsinfecdis.5c00225

**Published:** 2025-07-17

**Authors:** Martina M. Golden, Shehreen Siddiqui, Vivian Ohanaja, Savannah J. Post, William M. Wuest

**Affiliations:** † Department of Chemistry, 221304 Emory University Atlanta, Atlanta, Georgia 30322, United States; ‡ Antibiotic Resistance Center, Emory University School of Medicine Atlanta, Atlanta, Georgia 30322, United States

**Keywords:** *Pseudomonas aeruginosa*, sensitization, vancomycin, metabolism, clinical isolates

## Abstract

The global antibiotic resistance crisis causes nearly 5 million deaths annually. , a virulent Gram-negative bacterium, is a major cause of hospital-acquired infections, often coexisting with. Previous studies showed can be sensitized to vancomycin through altered nutrient availability. This study explores the scope and mechanisms of this phenomenon using a dual-pronged approach focused on primary metabolism. Through the application of a tool compound that targets succinate dehydrogenase, we sought to correlate this sensitization to effects seen in minimal media growth. Carbon supplementation can partially restore tolerance with sources that aid in detecting environmental changes, low iron levels, and altered metabolism. Vancomycin sensitization was also observed in multidrug-resistant clinical isolates, indicating that compensatory mutations may influence antibiotic susceptibility and metabolic flux. Our findings show that can also be sensitized to other gram-positive-specific antibiotics, such as erythromycin, chloramphenicol, and amoxicillin, with no apparent correlation to the antibiotic’s size or mechanism. These findings highlight how different growth conditions affect the susceptibility of to clinically relevant antibiotics.

## Graphical abstract

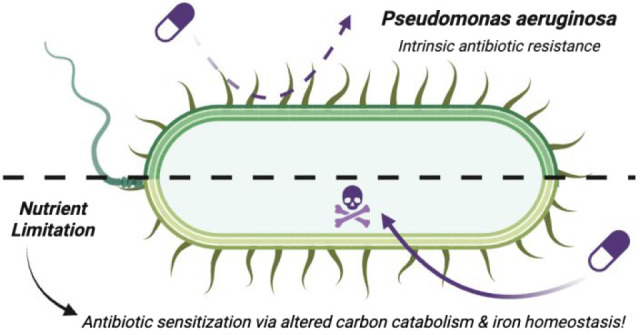

Antimicrobial resistance (AMR) is the ability of organisms, and in this case, bacteria, to survive antibiotic treatments. AMR affects people of all backgrounds and social classes worldwide and contributed to 4.95 million deaths in 2019.^1^ The evolution and development of resistance mechanisms in bacteria are sped up by frequent use or misuse of antibiotics, rendering these treatments ineffective over time.^2^ To combat antibiotic resistance, derivatives of antibiotics are created to inhibit bacterial growth in infections by bypassing resistance mechanisms. However, no new antibiotic classes have been discovered since the late 1900s.^3^ The discovery void has raised concerns about the increasing threat of AMR. Primarily, the ESKAPE pathogens (, , , , , and *Enterobacter)* have been identified as immediate public health threats to rising antibiotic resistance incidence due to their ability to quickly mutate, develop resistant mechanisms, and populate clinical settings. Sensitization of these bacteria could diminish their resistance mechanism's effectiveness.^4,5^

 is a highly virulent, gram-negative bacterium known for its ability to cause a wide range of infections in both immunocompromised and healthy individuals.^6^ is intrinsically resistant to many antibiotics because of its multidrug efflux pumps that can export antibiotics out of the cell and outer membrane porins that regulate the permeability of the outer membrane.^7^ In addition, can rapidly develop antibiotic resistance due to its high mutation rate and genetic plasticity.^8^ Its ability to develop broad-spectrum resistance is a significant challenge to combat in clinical settings, particularly in treating chronic infections such as those seen in cystic fibrosis patients, burn victims, and individuals with compromised immune systems.^9^ Moreover, readily adapts its metabolism to develop a successful infection, and metabolically deactivated cells are implicated in chronic infections as traditional antibiotics struggle to target these populations.

Antibiotic resistance in comes with an inherent fitness cost, meaning that resistant strains often experience a trade-off in terms of growth or virulence.^10–12^ Developing resistance to antibiotics often requires the bacterium to invest energy into mechanisms such as the efflux pumps, enzyme production, or alterations to the cell wall, which may limit resources available for other cellular functions.^13^ However, this cost is not always apparent in the short term, as resistant strains can evolve compensatory mechanisms over time to reduce the fitness burden associated with resistance. For example, ciprofloxacin-resistant isolates of can display collateral sensitivity to tobramycin and aztreonam.^14,15^ Investigations into this phenomenon are ongoing to determine the clinical applicability to address the ongoing AMR crisis.^16^

 is often found in association with in human infections.^17,18^ is a gram-positive bacterium that is treated with antibiotics such as vancomycin, amoxicillin, oxacillin, cephalexin, clindamycin, etc. Typically, displays a competitive advantage over and takes over as the infection progresses.^19,20^ Cystic fibrosis patients, in particular, are vulnerable to morbidity and mortality due to infection in adulthood that first starts as infection during childhood.^21,22^ This highlights the importance of studying how responds to antibiotic treatments for within the *in vivo* microenvironment of a coinfection. This is especially true within a multispecies biofilm, as they are known to be more resistant to antibiotics and experience stresses related to the limited resources within the biofilm. Moreover, it could potentially allow for one treatment to combat both and in bacterial infections that would be otherwise detrimental.

From 2003 to 2017, the Multidrug-Resistant Organism Repository and Surveillance Network (MRSN) associated with the Walter Reed Army Institute of Research has collected, sequenced, and characterized isolates of some of the most concerning pathogens, such as , , and .^23–25^ Within the panel, these strains exhibit a wide spectrum of antibiotic susceptibilities, ranging from pan-susceptible to pan-resistant, as well as diverse phenotypic traits, including distinct growth patterns and virulence factors. Continuous surveillance of multidrug-resistant (MDR) pathogens is essential for tracking emerging resistance trends, informing treatment strategies, and guiding antimicrobial stewardship efforts. By studying diverse clinical isolates of MDR pathogens, researchers can better understand resistance mechanisms and develop new therapeutic strategies to combat them. Moreover, it allows for further investigation of the fitness costs associated with AMR, potentially leading to novel molecular insights.

Our lab has been interested in studying the natural product promysalin as a tool compound, because of its selective inhibition of versus others in the genus. It does this through the inhibition of succinate dehydrogenase, which is conditionally essential in .^26–28^ Given this unique inhibitory activity, we hypothesized that it could be used in combination with clinical antibiotics to increase their efficacy against . This was not the case for many of the antibiotics tested, indicating a significant connection between bacterial metabolism and antibiotic efficacy. To our surprise, the only antibiotic that showed a synergistic relationship with promysalin was vancomycin, a gram-positive antibiotic. In the literature, it is established that vancomycin is not effective against gram-negative bacteria due to their highly impermeable outer membrane, but we found that membrane permeabilization was not a significant factor for the efficacy of our combination. This phenomenon aligns with the findings from Chan et al., who demonstrated sensitization to vancomycin under nutrient-limited conditions, independent of membrane permeability.^29^ Interestingly, was specifically sensitized to vancomycin in comparison to other glycopeptide antibiotics, adding to the mystery of the sensitization mechanism. We saw a connection between our studies given that promysalin targets primary metabolism and could induce a perturbation to metabolic flux similar to nutrient limitation. We hypothesized that there could be a similar underlying mechanism of sensitization between promysalin treatment and nutrient limitation, and it could have broader impacts beyond the laboratory.

In addition to understanding the mechanism of sensitization, we questioned whether resistant clinical isolates of obtained from the MRSN would be more susceptible to metabolism inhibition due to the variable fitness costs associated with antibiotic resistance. If multidrug-resistant strains are more susceptible to metabolism inhibition, it would stand that there could be enhanced sensitization to gram-positive antibiotics. These strains would allow us to test our hypothesis of whether fitness costs associated with resistance would enhance the potency of our natural product metabolism inhibitor or gram-positive targeting antibiotics. Moreover, it would determine whether this is a phenomenon that could have meaningful clinical implications when considering multispecies infections, such as those found in patients with cystic fibrosis.

## Results and Discussion

### Expanding the Scope of Gram-Positive Antibiotic Sensitization

To begin this study, we modified the nutrient limitation conditions presented by Chan et al. to develop reproducible minimum inhibitory concentrations (MICs) in our hands. When using LB, our vancomycin MICs would drift from 16 to 64 μg/mL. We employed cation adjusted MHB instead of the reported LB to take advantage of the steady MHB composition and endorsement from the Clinical and Laboratory Standards Institute (CLSI) for reproducible results.^30–32^ Consequently, we consistently observed the reported vancomycin MIC of 16 μg/mL (Figure S2). With robust growth conditions in hand, we screened growth in limited media against a diverse panel of gram-positive antibiotics (Table 1). The antibiotics selected varied in their mechanisms of action, classes, and size to delineate any obvious trends. In nutrient-limited conditions, is sensitized not only to vancomycin but also to amoxicillin, erythromycin, and chloramphenicol. Previously, (PAO1) has been

**Table 1:** Minimum Inhibitory Concentrations of Gram-Positive Antibiotics in (PA14 and PA14-Δ8) Grown in 100% MHB and 10% MHB^a^

Antibiotic	Class	Size (Da)	PA14–100%	PA14–10%	Δ8–100%	Δ8–10%
Vancomycin	Glycopeptide	1449	>256	16	>256	16
Amoxicillin	Penicillin	365	>256	8	>256	128
Oxacillin	Penicillin	401	>256	>256	256	128
Penicillin G	Penicillin	334	>256	>256	>256	>256
Cephalexin	Cephalosporin	347	>256	>256	>256	>256
Linezolid	Oxazolidinone	337	>256	>256	256	128
Chloramphenicol	-	323	256	32	1	1
Erythromycin	Macrolide	734	256	32	4	1
Clindamycin	Lincosamide	425	>256	>256	>256	>256
Bacitracin	AMP	1423	>256	>256	>256	>256
Daptomycin	AMP	1620	>256	>256	>256	>256
Rifampin	Rifamycin	823	>256	>256	>256	>256
Tetracycline	-	444	32	8	0.5	0.25
BAC	QAC	340	32	8	2	1

a AMP: antimicrobial peptide; QAC: quaternary ammonium compound.

shown to be more sensitive to chloramphenicol and tetracycline when treated in nutrient-deplete saline in comparison to rich media.^33^ Interestingly, was not sensitized to oxacillin, penicillin G, and cephalexin, which utilize the same mechanism of action as amoxicillin; a similar trend was observed for erythromycin and clindamycin. Moreover, we did not observe sensitization to daptomycin or bacitracin, which are similar in size to vancomycin. These data suggest that the mechanism of sensitization to gram-positive antibiotics is more nuanced than being confined to a particular size or class of antibiotic. This agrees with the work from Chan et al. where they found vancomycin to be the only glycopeptide that could be sensitized to, in addition to other large antibiotics. Additionally, this expands the scope of clinical antibiotics that could be sensitized to when in nutrient-limited conditions.

Upon testing the lab strain of , we also wanted to understand the role of efflux pumps in the sensitization of gram-positive antibiotics. Previously, our lab obtained an efflux deficient strain of PA14 wherein all eight of the major efflux pumps have been deleted. We hypothesized that this could inform whether efflux was involved in the mechanism of sensitization for gram-positive antibiotics. In PA14- Δ8, we observed inhibition of growth with oxacillin and linezolid which is consistent with previous reports of it being rapidly effluxed from . Moreover, we observed a significant decrease in the MIC of chloramphenicol and erythromycin, suggesting that these antibiotics are also subject to efflux. Given the large decrease in MIC between WT and Δ8 for erythromycin and chloramphenicol, it suggests that efflux is an important factor for the lack of activity in WT strains.^34^ In the 10% media, there is a change in the MIC for the WT but not the Δ8, therefore nutrient limitation could be limiting the efficacy of efflux pumps in WT PA14. Notably, we did not observe a change in the MIC of vancomycin in the Δ8 strain suggesting efflux is likely not involved in the tolerance mechanism.

### Nutrient-Dependent Vancomycin Sensitization Is Not the Result of Outer Membrane Permeability

We sought to further understand the mechanism of gram-positive antibiotic sensitization and determined that the outer membrane was a reasonable place to begin our investigations. To study the outer membrane, we chose to use divalent cations and small molecule probes to determine membrane permeability. Divalent cations such as Mg^2+^ and Ca^2+^ are crucial to the outer membrane integrity of gram-negative pathogens such as . Importantly, they are important for exclusion of many small molecules such as gram-positive antibiotics. Since we are in a minimal media, we hypothesized that potentially there was a deficiency of divalent cations, thus the exclusionary properties of the outer membrane would be compromised. We supplemented increasing concentration of Mg^2+^ and Ca^2+^ independently and observed that Ca^2+^ antagonized inhibition of growth more strongly than Mg^2+^, however, this was at the highest concentration of cation tested, which is far beyond the range of CA-MHB (Figure S3). Importantly, at the relevant concentrations of divalent cations, significant growth inhibition was observed for chloramphenicol and vancomycin. We considered that the cations could have a beneficial interaction when in combination. As such, we supplemented both cations to see how that would change the sensitization. In combination, the cations restored tolerance to amoxicillin (Figure 1B), suggesting that it could have exploited the deficient outer membrane to inhibit growth. The antagonistic effect of the cations was less obvious for chloramphenicol and erythromycin (Figure 1C,D). Interestingly, we observed inhibition of growth with 64 and 128 μg/mL vancomycin when the cations are supplemented to 100% levels (16–32 μg/mL) suggesting that this is not the mechanism of vancomycin sensitization (Figure 1A).

**Figure 1:**
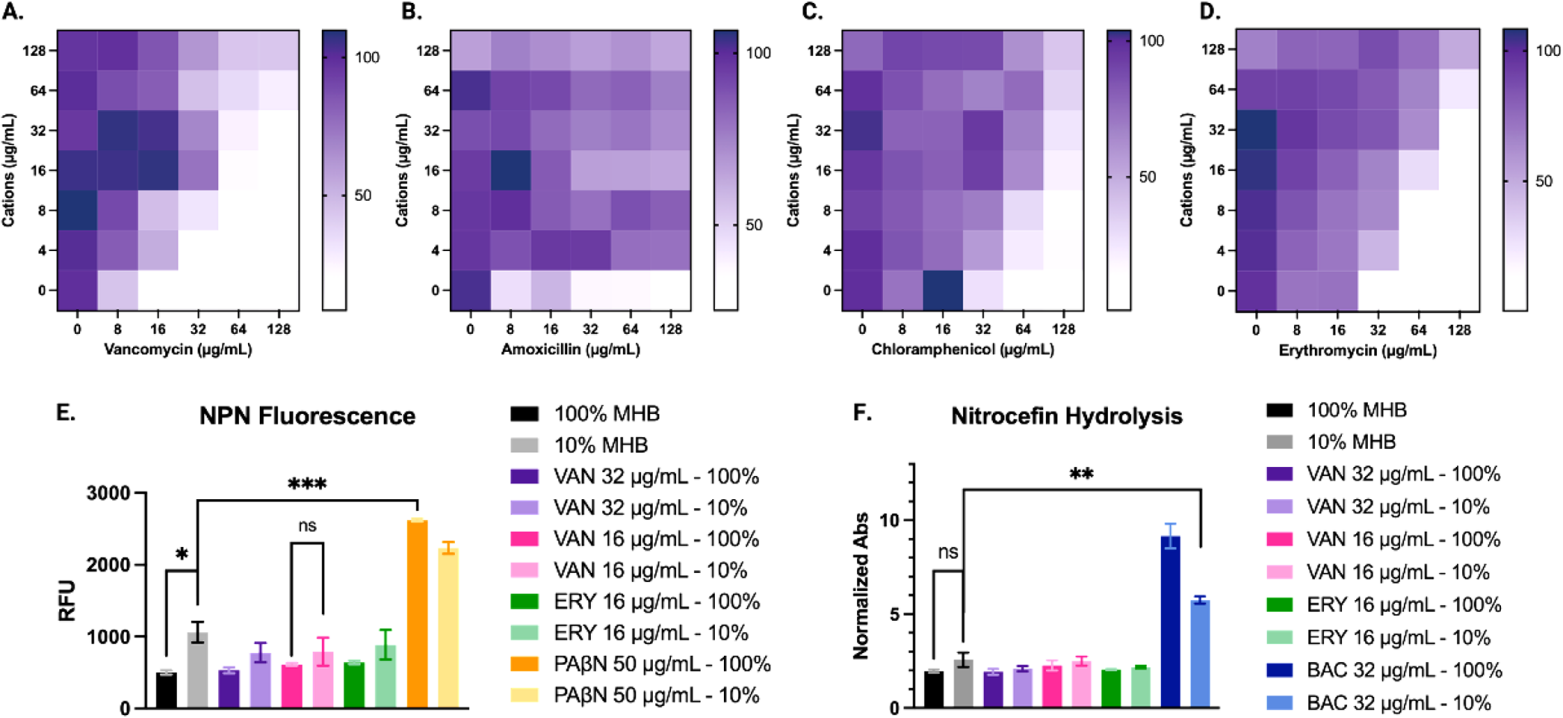
Outer membrane experiments in sensitized PA14. Antibiotic antagonism with divalent cation supplementation showing the normalized growth of the culture. (A) Vancomycin, (B) amoxicillin, (C) chloramphenicol, (D) erythromycin. Outer membrane permeabilization experiments using small molecule probes. (E**)** *N*-(1-Naphthyl)­aniline (NPN), (F) Nitrocefin. VAN: vancomycin; ERY: erythromycin; PAβN; phenylalanine-arginine β-naphthylamide; BAC: benzalkonium chloride. *Indicates *p*-value <0.05, ** *p*- value <0.005, and *** *p*-value <0.001, ns: not significant.

We sought to study the outer membrane of the sensitized cells using permeability probes. *N*-(1-Naphthyl)­aniline (NPN) is a hydrophobic small molecule that has low fluorescence in aqueous media, such as the extracellular environment, but will strongly fluoresce in a hydrophobic setting, such as the phospholipid bilayer. It was reported from Chan et al. that cells in 10% LB media were not permeabilized,^29^ therefore it would stand that changing the source of rich media would have minimal effect. As such, there was a slight increase in fluorescence for the cells grown in 10% MHB, but there was not a significant difference in the cells treated with vancomycin (16 or 32 μg/mL) or erythromycin (Figure 1E). Importantly, we employed Phenylalanine-arginine β-naphthylamide (PAβN) as a positive control as it has been shown to increase vancomycin inhibition in : specifically, 50 μg/mL of PAβN was required to achieve an MIC of 32 μg/mL of vancomycin.^35^ The fluorescence observed at this concentration of PAβN is vastly different from the fluorescence of 10% MHB cells, which can be correlated to the permeability of their outer membrane. These data suggest that the limited permeabilization observed in cells grown in 10% MHB is not responsible for vancomycin sensitization.

To support our outer membrane assay utilizing NPN, we sought to use the chromophore β-lactam, nitrocefin, as an additional outer membrane probe. Nitrocefin is a small molecule that cannot permeate the outer membrane of gram-negative bacteria, however, when the outer membrane is permeabilized, β-lactamases will readily hydrolyze it, thus changing the absorbance of the molecule from yellow (380 nm) to red (490 nm). In this assay, there was not a significant difference between the media conditions nor antibiotics (Figure 1F). These results support our conclusion that vancomycin sensitization, specifically, is not solely the result of a permeabilized outer membrane. This suggests that there’s a more nuanced mechanism of sensitization to be uncovered.

### Investigating the Mechanism of Vancomycin Sensitization

We were interested in studying whether there was a time-dependent aspect to vancomycin sensitization. Interestingly, we found that concentrations as low as 4 μg/mL of vancomycin inhibited the growth of cultures grown in 100% MHB (Figure 2A). Additionally, after 20 h, the cultures grown in 256 μg/mL vancomycin in 100% MHB begin to recover their growth. This suggested that is inherently somewhat susceptible to vancomycin, which has not been described in the literature previously. More recently, it was reported that vancomycin treatment exerts a strong selection pressure for the loss of flagella.^36^ To understand if nutrient limitation would exacerbate the selection pressure, we measured swimming ability after vancomycin treatment using a minimal swimming agar described by O’Toole et al.^37^ We did not observe a significant difference in the swimming ability of cultures grown in 100% media, regardless of vancomycin treatment (Figure 2B). However, there was a significant decrease in swimming for the cultures grown in 10% MHB that were treated with vancomycin. This is contrary to previous findings that report increased motility from cells under nutrient stress.^38^ Flagellum deficient has been shown to be more virulent in murine CF models.^39^ As mentioned above, cocultures of and are common in CF pulmonary infection, and vancomycin is the antibiotic of choice for methicillin-resistant .^40,41^ Additionally, this shows that vancomycin is affecting cells in sensitized conditions and rich media; however, the mechanism of entry remains undefined.

**Figure 2:**
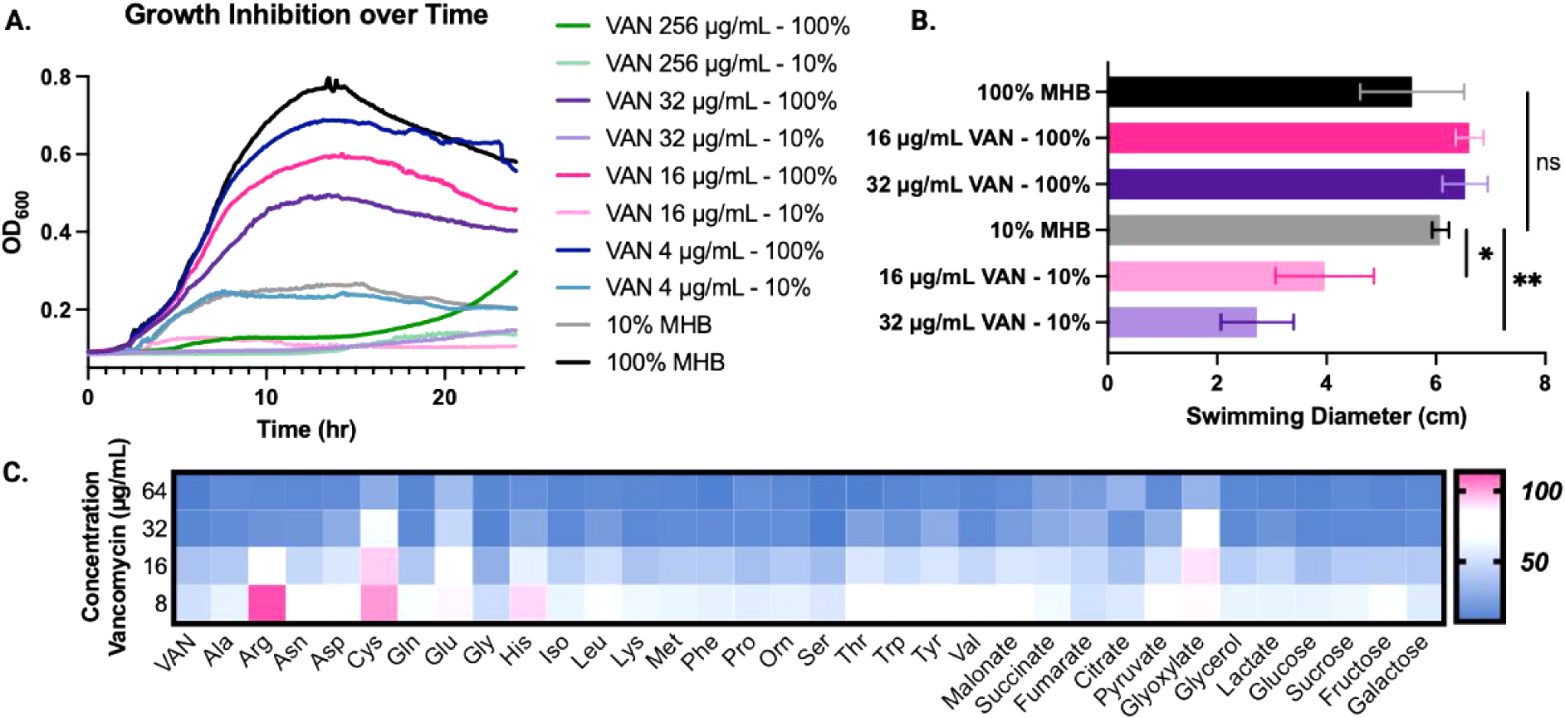
(A) Growth inhibition of (PA14) over 24 h with varying concentrations of vancomycin (256–4 μg/mL). (B) Swimming diameters for cultures grown in 100% and 10% media, with or without vancomycin treatment. ns: not significant. Representative images for these data are provided in Figure S3. (C) Heat map of 2.5 mM carbon supplementation of cultures treated with varying concentrations of vancomycin (64–8 μg/mL). Data normalized to media (0%, blue) and the negative control (100%, pink). At MIC (16 μg/mL): Arg (****), Cys (****), Glu (****), His (*), glyoxylate (****). All others are not significant. At 16 μg/mL: Arg (**), Cys (****), Glu (*), His (*), glyoxylate (*). *Indicates *p*-value <0.05, ** *p*- value <0.005, *** *p*-value <0.001, and **** *p*-value <0.0001, all others are not significant.

As a thought experiment, we examined all canonical mechanisms of antibiotic entry into the cell. The absence of significant permeabilization in our data suggests that this is not the mode of entry. The porins of have strict substrate specificities and generally do not accommodate molecules greater than 600 Da, suggesting that this would not be likely.^42–44^ Another potential option is oligopeptide transporters given the peptide backbone of vancomycin, however, the extensive cross-linking that rigidifies the structure would hinder uptake through this pathway. strains with genetically modified oligopeptide transporters are still resistant to vancomycin, supporting this conclusion.^45^ Finally, we considered a mechanism similar to the ionic interactions that assist aminoglycosides through the outer membrane.^2,46^ If this were the case, we would expect the MIC of aminoglycosides to decrease in 10% MHB, however, we did not observe a significant change to the MIC of tobramycin, gentamicin, or amikacin in 10% MHB (Table S2).

Given our data, we are confident that outer membrane permeabilization is not the mechanism of sensitization for vancomycin. Specifically thinking about metabolism, we wanted to determine whether general disruption was causing sensitization or if it was specifically related to carbon metabolism, pulling from our previous results with the tool compound promysalin. Carbonyl cyanide 3-chlorophenylhydrazone (CCCP) is a protonophore and thus disrupts cell metabolism indiscriminately. If CCCP synergizes with vancomycin, it would support the hypothesis that general metabolism disruption sensitizes to vancomycin. However, when treated together with cells grown in 100% MHB, there was no effect on the growth. This suggests that sensitization is specifically related to altered carbon metabolism, as we see with promysalin treatment (Figure S4).

To ascertain which carbon sources could be implicated in this phenomenon, we employed feeding studies with the hypothesis that specific carbon sources could decrease sensitivity toward vancomycin, and the identity of the carbon source would inform a potential mechanism of sensitization. Recently, it was shown that supplementing growth media can increase the susceptibility of a pathogen to antibiotics by promoting the pathways involved in the antibiotic mechanism of action or uptake.^47^ We supplemented carbon sources into the growth medium, ranging from amino acids, sugars, and metabolites. Starting with a supplement of 10 mM, we observed a significant increase in vancomycin tolerance and overall growth in 10% MHB with the addition of histidine (Figure S6). Several of the supplements inhibited growth at 10 mM so they were diluted 4-fold (2.5 mM) where no inhibition was observed in controls. Again, we supplemented carbon sources to sensitized cells exposed to varying concentrations of vancomycin. At the MIC (16 μg/mL), we observed several amino acids (Arg, Cys, Glu, His) show a significant increase in growth in the presence of vancomycin (Figure 2C). Arg and His are valuable sources of carbon and nitrogen for *Pseudomonas* and are implicated in detecting chemical signals from their environment, which could rationalize how they restore vancomycin resistance.^48–50^ The biosynthesis of pyochelin, specific siderophore, requires two molecules of Cys and could support the observation of strong promotion of growth in nutrient-limited media.^51^ Previously, it was shown the supplementation of iron into the dilute media antagonizes vancomycin sensitization.^29^ Combined with our results, this would suggest that the dilute media has insufficient iron concentrations to resist vancomycin treatment.

Surprisingly, succinate, the preferred carbon source for sp., did not rescue growth with vancomycin present.^52^ Additionally, we found that the only metabolite to elicit a significant response was glyoxylate. This was an unexpected finding as this is the metabolite for the shunt pathway of the TCA cycle, which we have found to be an unfavorable avenue for and thus rationalizes promysalin’s species selectivity due to this shortcoming. Studies have shown that in nutrient limitation, specifically for iron, will downregulate the iron-dependent enzymes of the TCA cycle to allocate resources more efficiently.^53^ However, the two enzymes in the glyoxylate shunt pathway, isocitrate lyase and malate synthase, are not iron dependent, suggesting that this pathway becomes more favorable in deficient environments. This could also connect promysalin treatment to nutrient limitation by shuttling carbon through an unpreferred pathway, thus limiting overall energy production.

### Sensitization of Antibiotic-Resistant Clinical Isolates

With the library of isolates in hand, we wanted to evaluate how they would respond to promysalin treatment. For these experiments, we chose to employ the structurally simplified promysalin analog (2), which differs only in the α-hydroxyl and maintains potent inhibition toward laboratory (PA14) (Figure 3A). In the environment, as bacteria acquire antibiotic resistance, there’s a fitness cost that arises with it. For example, energy is being spent producing antibiotic-degrading enzymes instead of supporting intracellular functions. Frequently, this is shown as a growth defect when culturing the bacteria, either their maximum cell density is lower, or it takes longer for them to reach maximum cell density. Because of this, we hypothesized that some of the strains in the library would be more susceptible to promysalin treatment than others. We selected a panel of strains ranging in their antibiotic resistance profiles to determine their susceptibility to promysalin (Figure 3B). We found that the clinical isolates were less susceptible than PA14, but some were similarly susceptible to 2 in comparison to PAO1. The most susceptible strain was MRSN 6220, which is a pan-resistant isolate. Notably, another pan-resistant strain (MRSN 5519) also shows increased susceptibility to metabolism inhibition by 2. Moreover, some of the less susceptible strains also displayed less antibiotic resistance, such as MRSN 4841 and 11566. Some isolates did not follow this trend and showed variable responses to 2 that were independent of their antibiotic resistance profiles, and additional genetic profiling is needed to determine the nature of these responses. However, overall, our hypothesis that increased antibiotic resistance would impose a fitness cost that would increase sensitivity toward 2 was supported.

**Figure 3:**
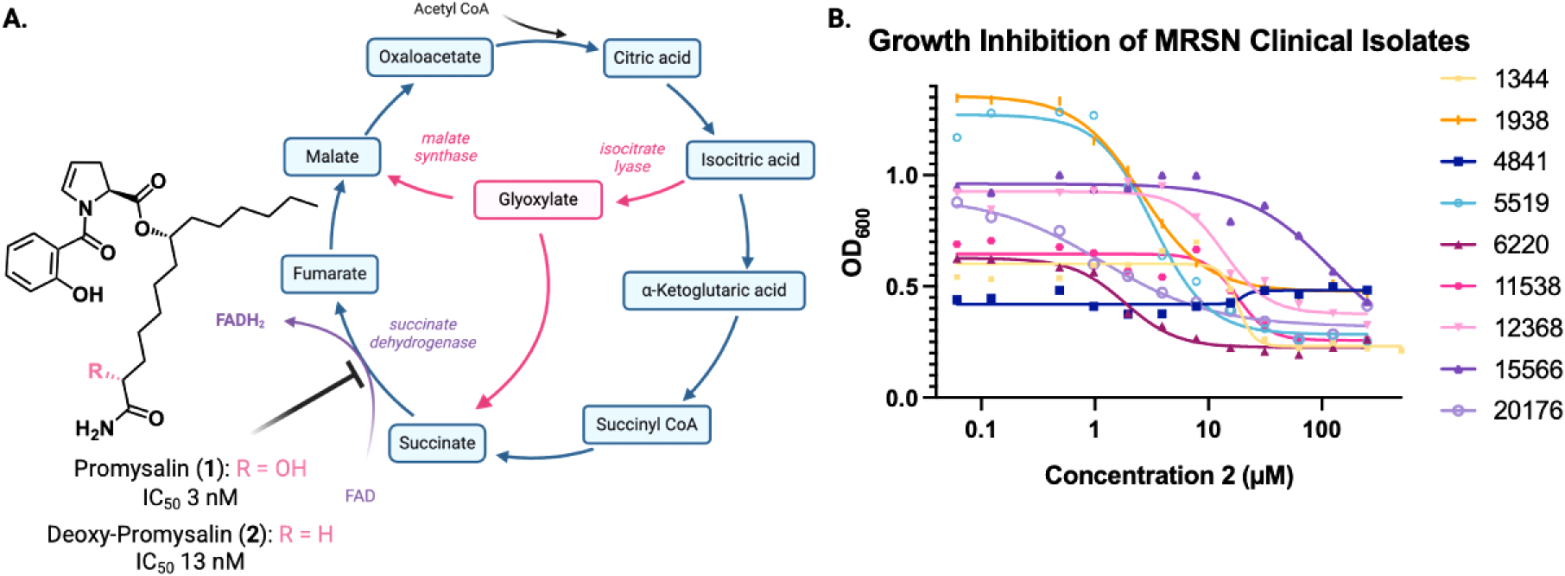
(A) Promysalin (**1**) and simplified analog (**2**) showing inhibition of succinate dehydrogenase in the tricarboxylic acid cycle (TCA cycle). (B) Growth inhibition of clinical isolates of from the Multidrug-Resistance Organism Repository and Surveillance Network (MRSN) using **2**.

Given that the clinical isolates from the MRSN exhibit varying susceptibilities to our metabolism inhibitor, promysalin, we hypothesized that they could also display differing susceptibilities to gram-positive antibiotics through collateral sensitivity. Moreover, we aimed to understand the clinical applications of nutrient limitation, as these populations are often found within biofilms, which are known to be nutrient-deficient environments.^54^ We hypothesized that some multidrug-resistant strains might experience collateral sensitivity to gram-positive antibiotics due to the high fitness cost associated with acquiring and maintaining antibiotic resistance genes.^55^ For this panel, we tested strains against vancomycin, amoxicillin, chloramphenicol, and erythromycin. We selected a diverse panel of strains from the MRSN library with various resistance patterns, growth capabilities, and susceptibility to promysalin. In addition to testing for promysalin susceptibility, we utilized strains resistant to trimethoprim and sulfamethoxazole, as folate synthesis is often tied to bacterial metabolism.^56^ For this, we examined MRSN strains 6220, 1906, 20176, and 12914; however, these did not increase their vancomycin sensitivity (Table S2). Seeing that antibiotic resistance did not correlate directly to vancomycin sensitization, we sought to assess whether differences in growth would influence a strain’s susceptibility to vancomycin. We selected strains 15566 and 5524 because they grew to lower-than-normal OD_600_ levels, while strain 1938 grew to nearly twice the OD_600_ of the lab strain PA14. Surprisingly, strain 1938 exhibited greater sensitivity to vancomycin than any of the clinical isolates tested thus far, with an MIC of 8 μg/mL in 10% MHB. In comparison, strains 15566 and 5524 had an MIC of 64 μg/mL in 10% MHB. Considering this, we investigated other isolates that displayed strong growth with a maximum OD_600_ exceeding 1.5. Of the seven that we tested, 5519 was particularly notable, with an MIC of 16 μg/mL, while the others had MIC exceeding this value.

The strains that are most sensitized to vancomycin are 5519 and 1938, which both happen to grow to high optical densities. Upon further inspection, the resistance genes that they have in common are *catB7*, *fosA*, and *sul1*. However, these genes are found in many of the strains we tested that were not sensitized to the same extent. Beyond the antibiotic resistance genes, there are likely compensatory mutations in these strains that are contributing to their vancomycin sensitization and warrant future studies. Thorough genomic analysis of these strains could elucidate novel mutations involved in antibiotic sensitization and provide a foundation for translational studies.

## Conclusion

 is a master at metabolic perturbations; however, it can be disturbed with a small molecule, promysalin, through inhibition of succinate dehydrogenase. Using a library of clinical isolates from the MRSN, we saw that strains that were more resistant to antibiotics were more susceptible to promysalin treatment. This suggests that the fitness cost associated with acquiring AMR disrupts ’s metabolic flexibility. Previous work showed that vancomycin synergy with promysalin is a unique phenomenon with results that were not replicated with other gram-positive antibiotics. However, there are greater implications to nutrient limitation than altered metabolism, which sensitized to other gram-positive antibiotics. With our efflux-deficient strain, we observed greater than 200-fold decreases in the MICs for erythromycin and chloramphenicol, suggesting that efflux is a major contributor to their lack of activity in . We hypothesize that when cells are cultured in 10% MHB, efflux pumps are not as active because they require energy to function. Importantly, there is no clear pattern in size or drug class of gram-positive antibiotics that can best predict inhibition.

To investigate the mechanism of sensitization, we performed outer membrane permeability assays with small molecules and divalent cation supplementation. We deduced that outer membrane permeability is not the primary mechanism for sensitization. Moreover, vancomycin appears negatively affect in 100% MHB which was previously not described in the literature. Carbon source supplementation revealed that some molecules, such as Arg, Cys, Glu, and His, can promote growth, which may point to certain metabolic pathways involved in the sensitization mechanism. Previously, it was shown that the addition of metals, such as iron, could antagonize vancomycin sensitization.^29^ This supports our hypothesis of cysteine contributing to siderophore biosynthesis to increase intracellular iron concentrations. However, the exact mechanism of vancomycin entry to remains elusive. RNA sequencing would help pinpoint transient changes in genes modulated in sensitized in response to vancomycin treatment. Furthermore, additional genomic analysis of MRSN isolates could clarify potential genetic factors that restrict metabolic flux, thereby predisposing to vancomycin sensitization. Building off our results, further details can be learned about the mechanism by which sensitization reduces resistance to vancomycin.

Clinically, understanding how responds to gram-positive antibiotics will provide critical insights into managing infections with cocultures. Moreover, this study highlights the importance of studying pathogens within a representative infection model to determine all potential interactions between antibiotics and bacteria. This could aid in bridging the gap between the laboratory and clinic or provide novel insights into how bacteria respond to nontraditional treatments. Through improved infection management, patients can experience lower mortality rates and better treatment outcomes for those with compromised immune systems or severe infections. Future work could expand the scope to other gram-negative pathogens and determine if there are unique conditions for which they are sensitized to new antibiotics. This could provide valuable information for developing the next generation of antimicrobials.

## Supplementary Material



## Notes

General information; chemicals and reagents; detailed biological procedures; minimum inhibitory concentration assays; supporting figures and tables (PDF)
