# Supplementary material for: Vancomycin Sensitization in is Contingent on Limited Metabolic Flux
Source: ACS Infect Dis. 2025 Jul 17;11(8):2169–77. doi: 10.1021/acsinfecdis.5c00225 (PMC12340952; doi:10.1021/acsinfecdis.5c00225)
Supplement: Supplementary file 1 [file id5c00225_si_001.pdf]

Supporting Information

Vancomycin sensitization in *Pseudomonas aeruginosa* is contingent on limited metabolic flux

Martina M. Golden[a], Shehreen Siddiqui[a], Vivian Ohanaja[a], Savannah J. Post[a], and William M. Wuest*[a,b]

[a] Department of Chemistry, Emory University, Atlanta, Georgia 30322, United States
[b] Emory Antibiotic Resistance Center, Emory University School of Medicine, Atlanta, Georgia 30322, United States

To whom correspondence should be addressed. E-mail: wwuest@emory.edu

Table of Contents

1. **Supporting Figures** **S2-S5**

Supplemental Figure S1: Growth inhibition of clinical isolates of *Pseudomonas aeruginosa* from the Multidrug-Resistance Organism Repository and Surveillance Network (MRSN) using **2**.

Supplemental Figure S2: Growth optimization of *P. aeruginosa* for sensitization to vancomycin.

Supplemental Figure S3: Divalent cation supplementation to PA14 grown in 10% MHB and treated with Gram-positive antibiotics.

Supplemental Figure S4: Representative images of *P. aeruginosa* swimming motility after vancomycin exposure with measurements annotated.

Supplemental Figure S5: 10 mM carbon supplementation to PA14 treated with vancomycin.

2. **Supporting Figures** **S6-S7**

Supplemental Table S1: Minimum inhibitory concentrations of Gram-positive antibiotics against clinical isolates of *P. aeruginosa* obtained from the Multidrug-Resistant Organism Repository and Surveillance Network (MRSN).

Supplemental Table S2: Minimum inhibitory concentrations of aminoglycosides against *P. aeruginosa* in 100% and 10% MHB.

3. **Materials & Methods** **S7-S9**

3.1. Bacterial strains and culture conditions
3.2. Minimum Inhibitory Concentration (MIC) assay
3.3. Checkerboard assay
3.4. NPN assay
3.5. Nitrocefin assay
3.6. Carbon supplementation
3.7. Swimming Motility

4. References S9

1. Supporting Figures and Tables

Supplemental Figure S1: Growth inhibition of clinical isolates of *Pseudomonas aeruginosa* from the Multidrug-Resistance Organism Repository and Surveillance Network (MRSN) using **2**.

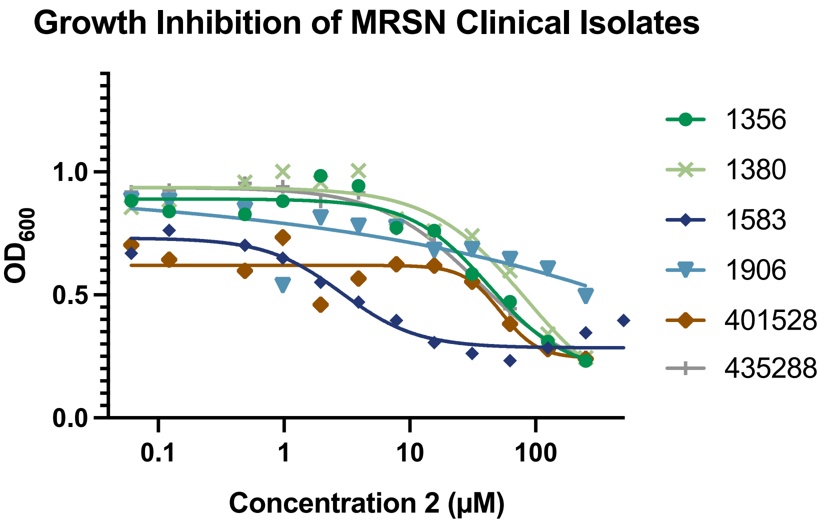

Supplemental Figure S2: Growth optimization of *P. aeruginosa* for sensitization to vancomycin.

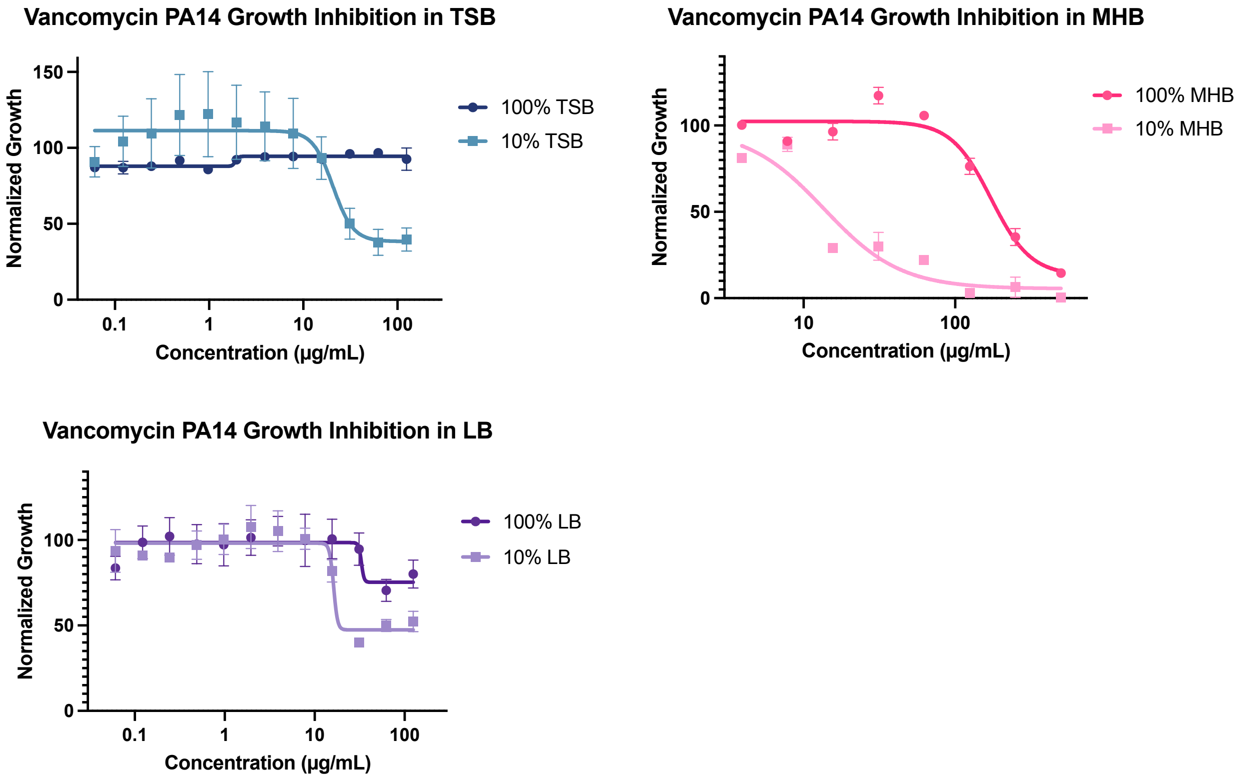

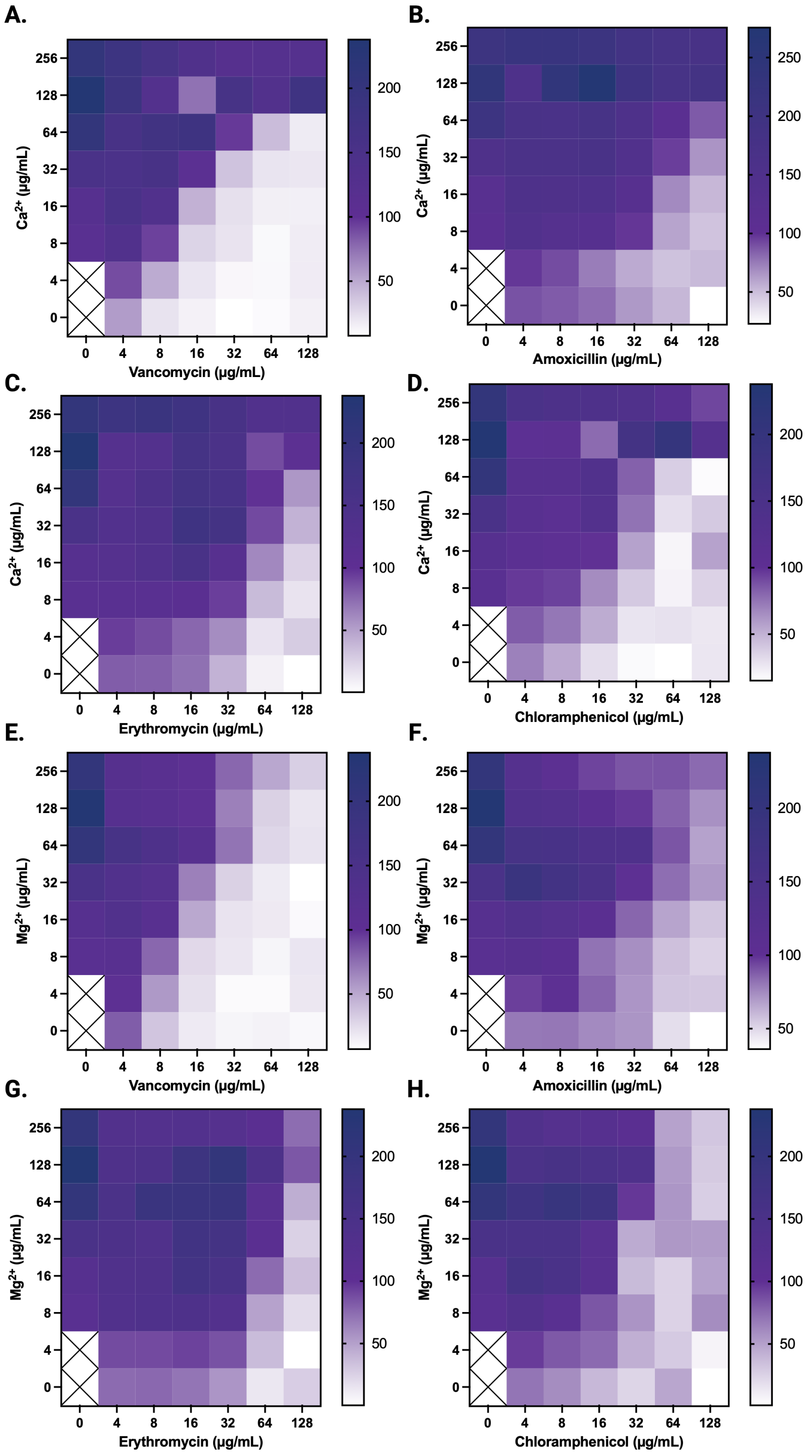
A.
B.
C.
D.
E.
F.
G.
H.
Vancomycin (μg/mL)
Amoxicillin (μg/mL)
Erythromycin (μg/mL)
Chloramphenicol (μg/mL)
Vancomycin (μg/mL)
Amoxicillin (μg/mL)
Erythromycin (μg/mL)
Chloramphenicol (μg/mL)
Ca^{2+} (μg/mL)
Mg^{2+} (μg/mL)
0 4 8 16 32 64 128
256 128 64 32 16 8 4 0

Supplemental Figure S3: Divalent cation supplementation to PA14 grown in 10% MHB and treated with Gram-positive antibiotics. Magnesium supplements: **A.** Vancomycin, **B.** Amoxicillin, **C.** Erythromycin, **D.** Chloramphenicol. Calcium supplements: **E.** Vancomycin, **F.** Amoxicillin, **G.** Erythromycin, **H.** Chloramphenicol. Data marked with an X was omitted but does not alter the qualitative analysis of the data.

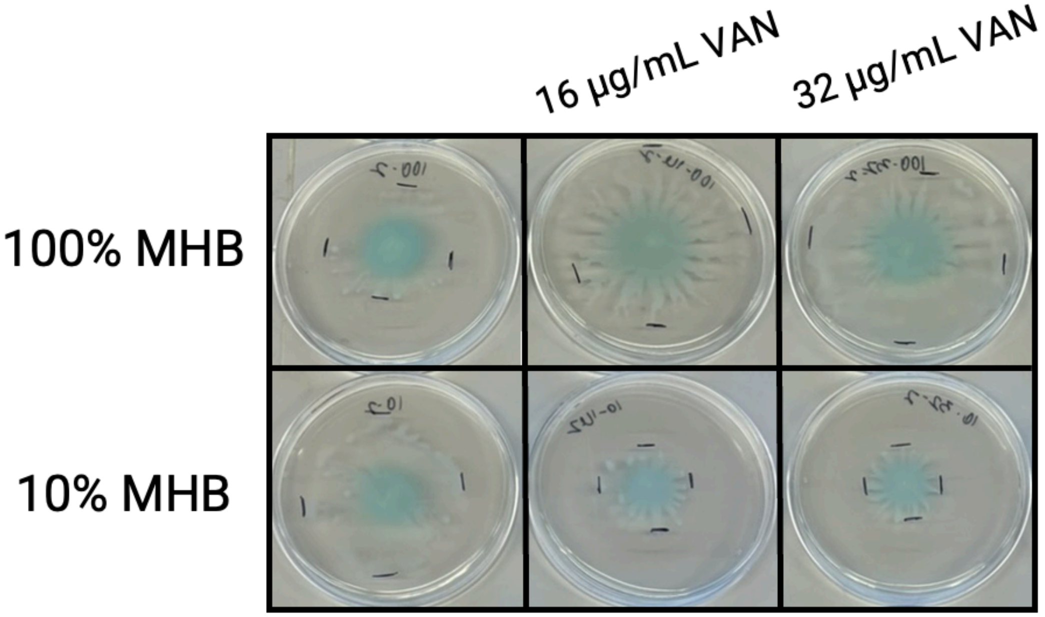

Supplemental Figure S4: Representative images of *P. aeruginosa* swimming motility after vancomycin exposure with measurements annotated.

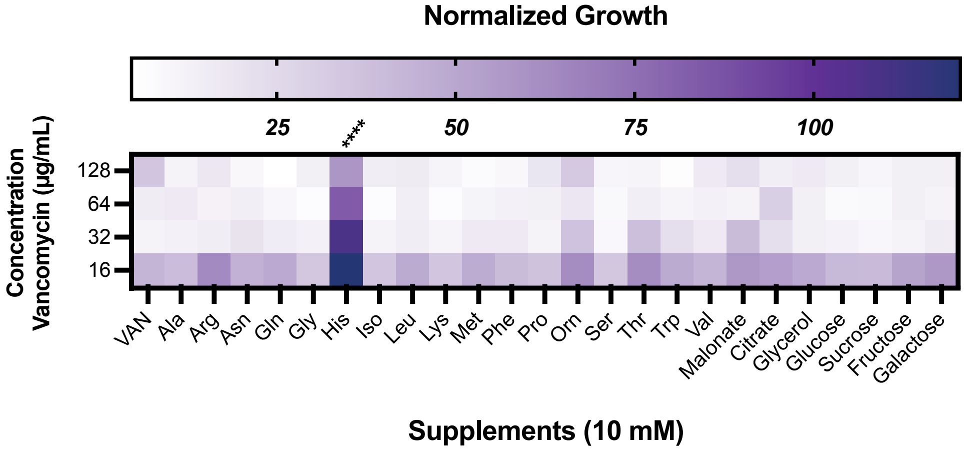

Supplemental Figure S5: 10 mM carbon supplementation to PA14 grown in 10% MHB and treated with vancomycin. ***Note:*** Aspartate, cysteine, glutamate, succinate, fumarate, pyruvate, glyoxylate and lactate inhibited PA14 growth in 10% MHB at 10 mM and have been excluded from the data above.

Supplemental Table S1: Resistance scores and minimum inhibitory concentrations of Gram-positive antibiotics against clinical isolates of *P. aeruginosa* obtained from the Multidrug-Resistant Organism Repository and Surveillance Network (MRSN). All strains were tested in 100% and 10% MHB. Resistance scores were determined based on data obtained from MRSN and represent the number of antibiotics the strain was resistant to out of the 16 tested (amikacin, ampicillin/sulbactam, ampicillin, aztreonam, cefazolin, cefepime, ceftazidime, ceftriaxone, ciprofloxacin, gentamicin, imipenem, levofloxacin, meropenem, piperacillin/tazobactam, tobramycin, trimethoprim). Half scores are indicative of intermittent resistance phenotypes. Amoxicillin has been omitted as all MICs (100% and 10% MHB) were 256 or > 256. Values are reported in µg/mL.

Note: A table describing the strain's full resistance profiles was published by Lebreton et al. Refer to the original publication for complete details.

		Vancomycin		Chloramphenicol		Erythromycin		Tobramycin		BAC	
**MRSN**	Resistance Score	100%	10%	100%	10%	100%	10%	100%	10%	100%	10%
**1906**	9	> 256	32	> 256	> 256	128	64	128	4	64	4
**1938**	10.5	> 256	8	> 256	256	256	64	32	2	128	8
**20176**	15	> 256	256	> 256	> 256	> 256	128	> 256	> 256	64	16
**6241**	15	> 256	32	> 256	128	256	128	16	32	64	4
**12914**	15	> 256	64	> 256	256	128	128	64	64	256	64
**6220**	16	> 256	32	> 256	> 256	256	128	256	64	32	8
**15566**	7.5	> 256	64	> 256	> 256	64	32	0.25	< 0.125	32	16
**5524**	13.5	> 256	64	> 256	64	256	128	32	8	256	8
**19711**	8.5	> 256	32	> 256	256	256	128	0.5	< 0.125	32	16
**12365**	7.5	> 256	32	64	64	256	64	2	0.25	64	32
**552**	6	> 256	32	> 256	256	256	128	0.5	< 0.125	64	32
**5519**	16	> 256	16	> 256	16	256	64	128	32	64	2
**994**	13	> 256	64	> 256	256	256	128	1	< 0.125	32	16
**11538**	10.5	> 256	32	> 256	> 256	256	256	0.25	< 0.125	32	16
**321**	10	> 256	32	> 256	64	256	128	0.25	< 0.125	32	16
**1344**	7	> 256	256	> 256	128	> 256	256	0.25	< 0.125	32	16
**4841**	11.5	> 256	128	> 256	256	128	256	2	0.5	64	16

Supplemental Table S2: Minimum inhibitory concentrations of aminoglycosides against *P. aeruginosa* in 100% and 10% MHB.

	**PA14**	
	**100% MHB**	**10% MHB**
**Gentamicin**	0.5	0.125
**Amikacin**	0.5	0.125
**Kanamycin**	128	64
**Tobramycin**	0.25	0.125

2. Materials & Methods

2.1. Bacterial strains and culture conditions

PAO1 was obtained from Prof. George O'Toole (Dartmouth University), and PA14 was acquired from Prof. Joanna B. Goldberg (School of Medicine, Emory University). The efflux-deficient strain (PA14-*Δ*8) was a gift from Genentech, as previously disclosed.[1] *P. aeruginosa* clinical isolates were obtained from the Multidrug-Resistant Organism Repository and Surveillance Network (MRSN).[2] All strains were streaked onto Mueller Hinton agar (MHA) from a freezer stock and incubated overnight (16–24 h). Then, single colonies were picked and grown overnight while being shaken at 37 °C at 200 rpm in cation-adjusted Mueller Hinton broth (CA-MHB) (5 mL). The overnight cultures were diluted 1:100 in 5 mL of fresh medium (5 mL CA-MHB or 5 mL 10% CA-MHB in PBS) and grown at 37 °C with 200 rpm shaking to mid-exponential growth as determined by the OD_{600} (optical density at 600 nm) reading (measured on a BioTek Synergy H1 hybrid plate reader). The bacteria were then diluted to a concentration of 0.004 according to the following equation: (x µL regrow culture) × (OD reading) = (0.004) × (volume of diluted bacteria culture needed).

2.2. Minimum Inhibitory Concentration (MIC) assay

The antibiotics were prepared in a 10.24 mg/mL DMSO stock and diluted 1:9 in water yielding 1,024 µg/mL stock dissolved in 10% DMSO in water. Then, the compounds were serially diluted 2-fold in water to produce 12 concentrations ranging from 256-0.125 µg/mL, with a final volume of 100 µL per well. Negative controls of compound vehicles were employed. Next, 100 µL of the diluted bacteria were plated into each well, and the plates were incubated statically for 24 h at 37 °C. The MIC was determined as the lowest concentration where there was no growth visible to the naked eye.

2.3. Checkerboard assay

One compound was serially diluted 2-fold in vehicle 11 times so there was no compound in the final column (11 concentrations). The second compound was added to all wells in the first 7 rows in decreasing concentrations. Water was added to bring final volume to 100 µL. 100 µL of diluted bacteria was plated into each well, then the plates were incubated statically for 24 hours at 37 °C. OD_{600} readings were taken at this point and growth was normalized to media control and negative control. The assay was tested in triplicate from three separate overnight cultures.

2.4. NPN assay

Overnight cultures of PA14 were diluted 1:100 in fresh media (5 mL CA-MHB or 5 mL 10% CA-MHB in PBS) and allowed to grow to mid-exponential phase (OD_{600} 0.5) at 37 °C with shaking at 200 rpm. Cells were harvested by centrifugation (3900 rpm, 25 °C, 10 min) and washed twice with 5 mM GHEPES buffer (5 mM HEPES and 5 mM glucose). Cells were resuspended in assay buffer to a final OD_{600} of 1. Then, 90 µL of cells were combined with 90 µL GHEPES with 20 µM NPN (from a 5mM stock solution of NPN in acetone) in a 96-well optical-bottom black plate and incubated for 20 minutes. Compounds were diluted and 10% DMSO concentration was maintained for all compounds. After 20 minutes of incubation, 20 µL of compound or vehicle was added to every well and fluorescence was immediately monitored at an exciting wavelength of 350 nm and emission wavelength of 420 nm for 30 minutes to 1 hour. The fluorescence will stabilize during this time. Maximum permeabilization (NPN uptake) was determined to be the maximum fluorescence intensity which was normalized to background NPN fluorescence.

2.5. Nitrocefin assay

PA14 cells grown overnight in CA-MHB and diluted 1:100 in fresh media (5 mL CA-MHB or 5 mL 10% CA-MHB in PBS) and grown at 37 °C with shaking to mid-exponential phase, centrifuged, washed in PBS, and resuspended to OD_{600} = 0.2 in 20 mM PBS with 1mM $MgCl_2$ at pH 7.2. A volume of 50 µL of the cell suspension was added to a clear, flat-bottom 96-well plate containing 50 µL of PBS with a final concentration of 50 µg/mL nitrocefin and the antibiotic of interest. Plates were incubated at 37 °C in a stationary incubator and absorbance was read for 60 min at 5 min intervals at 490 nm to monitor nitrocefin hydrolysis. Reads were normalized using the corresponding no cell control wells.

2.6. Carbon supplementation

PA14 cells grown overnight in CA-MHB and diluted 1:100 in fresh media (10% MHB) and grown at 37 °C with shaking to mid-exponential phase. A flat bottom 96-well plate was prepared with 50 µL vancomycin which will yield final concentrations of 8 µg/mL to 256 µg/mL. 50 µL of the supplement stock (40 mM or 10 mM) was added to the solution yielding a total volume of 100 µL. Bacteria were diluted in 10% MHB according to the above method and 100 µL of the diluted culture was added to the plate. The plates were incubated statically for 24 hours at 37 °C. OD_{600} readings were taken at this point, and growth was normalized to media control and negative control. The assay was tested in triplicate from three separate overnight cultures. Significance was determined with an unpaired t-test in GraphPad Prism 10.

2.7. Swimming motility

Cells were prepared according to the procedure above. This has been adapted from O'Toole *et al* with minor modifications indicated.[3,4] 100 µL of diluted vancomycin was added to a flat-bottom 96-well plate yielding concentrations of 16 and 32 µg/mL. 100 µL of diluted bacteria were added and the plates were incubated statically for 18 hours at 37 °C. Agar plates for assessing swimming motility and swarming motility consisted of M8 medium (6 g Na_2HPO_4, 3 g KH_2PO_4, and 0.5 g NaCl for 1 L) supplemented with glucose (0.2% w/v), $MgSO_4$ (1 mM), Casamino Acids (0.5 w/v), and 0.3% agar for swimming. 1 µL of stationary culture from each condition was inoculated into the agar and incubated for 18 hours at 37 °C. The swimming diameter was determined to be the average of two perpendicular measurements. The assay was tested in triplicate from three separate overnight cultures. Significance was determined with an unpaired t-test in GraphPad Prism 10.

References:

1. A. R. Mahoney, K. M. Storek, W. M. Wuest. Structure-Based Design of Promysalin Analogues to Overcome Mechanisms of Bacterial Resistance. *ACS Omega* 2023, 8 (13), 12558–12564.
2. F. Lebreton, E. Snesrud, L. Hall, et al. A panel of diverse Pseudomonas aeruginosa clinical isolates for research and development. *JAC Antimicrob Resist* 2021, 3 (4), dlab179.
3. D.G. Ha, S.L. Kuchma, G.A. O'Toole. Plate-Based assay for swimming motility in pseudomonas aeruginosa. *Methods in Molecular Biology* 2014, 1149, 59–65.
4. G.G. Nicastro, G.H. Kaihami, A.A. Pulschen, et al. c-di-GMP-related phenotypes are modulated by the interaction between a diguanylate cyclase and a polar hub protein. *Sci Rep* 2020, 10 (1), 1–11.
